# Ectopic Expression of *Litsea cubeba LcMADS20* Modifies Silique Architecture

**DOI:** 10.1534/g3.119.400611

**Published:** 2019-10-15

**Authors:** Yulian Jiao, Hengfu Yin, Yicun Chen, Ming Gao, Liwen Wu, Yangdong Wang

**Affiliations:** * State Key Laboratory of Tree Genetics and Breeding, Chinese Academy of Forestry, Beijing 10091, China and; † Research Institute of Subtropical Forestry, Chinese Academy of Forestry, Hangzhou 311400, China

**Keywords:** Lauraceae, mountain pepper, AGAMOUS-like genes, ectopic expression, fruit

## Abstract

*Litsea cubeba* (Lour.) Pers. (mountain pepper, Lauraceae) is an important woody essential oil crop that produces fragrant oils in its fruits, especially in its peels. Identification of genes involved in the regulation of fruits and peel architecture is of economic significance for *L. cubeba* industry. It has been well known that the MADS-box genes are essential transcription factors that control flowers and fruits development. Here, we obtained 33 MADS-box genes first from the RNA-seq data in *L. cubeba*, and 27 of these genes were of the MIKC-type. *LcMADS20*, an AGAMOUS-like gene, was highly expressed in the developing stages of fruits, particularly at 85 days after full bloom. The ectopic expression of *LcMADS20* in *Arabidopsis* resulted in not only curved leaves, early flowering and early full-opened inflorescences, but also shorter siliques and decreased percentage of peel thickness. Moreover, in the *LcMADS20* transgenic *Arabidopsis*, the expression modes of several intrinsic ABC model class genes were influenced, among which the expression of *FUL* was significantly reduced and *AP3*, *AG*, and *STK* were significantly increased. This study systematically analyzed the MADS-box genes in *L. cubeba* at the transcriptional level and showed that *LcMADS20* plays important roles in the regulation of fruit architecture.

*Litsea cubeba*, a dioecious species in the family Lauraceae, is regarded as an important industrial essential oil crop (Chen *et al.* 2013). *L. cubeba* essential oil, which is mainly extracted from the fresh fruits (especially from the peel), has been widely used for as botanical insecticides, natural additives, and ionone series flavors, in addition to other functions (Jiang *et al.* 2009; Agrawal *et al.* 2011; Wang *et al.* 2012; Su and Ho 2016). *L. cubeba* produces fragrant oils in its fruits, especially in its peels (Chen *et al.* 2013). Investigation of mechanisms involved in the regulation of fruits and peel architecture is specifically important for *L. cubeba* industry. However, studies on the growth and development of fruit and peel in *L. cubeba* remain rare. Generally, the majority fruit development begins after the ovary fertilization, and then the ovary wall differentiates into pericarp (endocarp, mesocarp, and exocarp) (Pabonmora *et al.* 2014). It has been well known that members of the MADS-box family genes encode for transcription factors essential in the control of flower and fruit development (Pinyopich *et al.* 2003; Lee and Lee 2010; Bowman *et al.* 2012). For example, the AG-like genes, which identity carpel, stamen and ovule, can affect fruit yield and quality (Pinyopich *et al.* 2003; Losa *et al.* 2010).

The MADS-box genes were initially identified from four founding proteins (Mcm1, Agamous, Deficiens A, Serum response element) from yeast (*Saccharomyces cerevisiae*), *Arabidopsis thaliana*, *Antirrhinum majus*, and mammals (human) respectively (Schwarz-Sommer *et al.* 1990). These proteins all have a contiguous conserved sequence of approximately 58 amino acids, which includes dimerization and a DNA binding domain (Schwarz-Sommer *et al.* 1990). In *Arabidopsis*, the MADS-box genes are classified into five groups named Mα, Mβ, Mγ, Mδ, and MIKC (Parenicová *et al.* 2003). Members of the MADS-MIKC group, which play important roles in flower development, include the M, I, K, and C domains (Causier *et al.* 2010). In general, the most conserved M domain is responsible for DNA binding and protein dimerization; the I intervening domain participates in dimerization specificity; the K domain is responsible for protein-protein interaction; and the C domain plays an essential role in specific functions (Causier *et al.* 2010; Kramer *et al.* 1998).

The MADS-box genes play variety functions in flower and fruit architecture. The flowering time regulators *FLOWERING LOCUS C (FLC*), *SHORT VEGETATIVE PHASE* (*SVP*), and *SUPPRESSOR OF OVEREXPRESSION OF CONSTANS1* (*SOC1*) can integrate numerous environmental and endogenous signals to regulate flower formation and development (Searle *et al.* 2006; Lee *et al.* 2007; Lee and Lee 2010). Moreover, the floral ABC model genes function in flower organ identity and formation (Theissen and Saedler 2001; Causier *et al.* 2010). The A class gene, *APETALA1* (*AP1*), can control sepal identity and interacts with the B class genes *PISTILLATA* (*PI*) and *APETALA3* (*AP3*) involved in the control petal identity. The C class gene *AGAMOUS* (*AG*) is a carpel identity gene and is necessary for stamen development, together with the B class genes (Causier *et al.* 2010; Smaczniak *et al.* 2012). The MADS-box transcription factors *SEEDSTIC*K (*STK*), *SHATTERPROOF1* (*SHP1*), and *SHATTERPROOF2* (*SHP2*) belong to the D class genes and play a redundant role in controlling ovule integument identity (Pinyopich *et al.* 2003; Losa *et al.* 2010). The E class genes *SEPALLATA1–4* (*SEP1–4*) are required for the specification of all four types of floral organs (Pelaz *et al.* 2000; Ditta *et al.* 2004).The MADS-box genes can also interact with the family genes and also with non-MADS transcriptional regulators or cofactors (Brambilla *et al.* 2007; Smaczniak *et al.* 2012).

*AG* is necessary for development of the reproductive organs (stamens and carpels). The loss of *AG* function caused homeotic conversion fromstamens and carpels to petals in *Arabidopsis* (Bowman *et al.* 1989), the overexpression of *AG* caused curled leaves, staminoid petals, carpelloid sepals, and inflorescence in *Arabidopsis* (Riechmann and Meyerowitz 1997). In rice (*Oryza sativa* L.), ectopic expression of *OsMADS3*, an AG-like gene, caused homeotic conversion of lodicules to stamens (Kyozuka and Shimamoto 2002). The redundant functions of *AG* and the *SHP* genes can promote carpel development, and there are redundant roles of *AG*, *STK*, and *SHP* genes in ovule identity (Pinyopich *et al.* 2003). Ectopic expression of either the *STK* or *SHP* gene is sufficient to induce the transformation of sepals into carpeloid organs bearing ovules (Favaro *et al.* 2003). To investigate the important roles of MADS-box transcription factors involved in the flower and fruit development in *L. cubeba*, we first identified the MADS-box genes in *L. cubeba* and then conducted a functional analysis on the AGAMOUS-like gene *LcMADS20*. This work was estimate to provide important information of genes involved in the fruit architecture for strategy of *L. cubeba* plant industry.

## Materials and Methods

### Plant materials

The plant material of *L. cubeba* used in this paper is the family of Anhui5^#^. *L. cubeba* is a dioecious plant, usual flowers in March, and full blooms at the middle of March. The pollen of the flower deteriorates in the female tree, while the ovary of the flower deteriorates in the male tree (Figure S1). After the flower fertilized in the female tree, the fruits begin to develop, and usually matured in August. To conduct the analysis on LcMADS-box genes and the expression mode during the three different developmental stages of flower bud, we used our previously published RNA-seq data for the three stages, which included the first stage (FD1, MD1) (the inflorescence primordium begins to differentiate), the second stage (FD2, MD2) (the flower primordium begins to differentiate) and the third stage (FD3, MD3) (the flower organ begins to differentiate) (He *et al.* 2018). The fruit samples for RT-qPCR picked on 25, 40, 55, 70, 85, 100, and 115 DAF (Days After Full bloom). All samples immediately frozen in liquid nitrogen and then stored at –80° until the RNA extraction. *Arabidopsis* of wide type (Columbia) used in this study.

### Identification of MADS-box genes in L. cubeba

The HMM (Hidden Markov Models) profiles of the MADS domain (PF00319) were acquired from the PFAM database (Finn *et al.* 2016) and then searched against the RNA-seq translated coding sequence with HMMER software (3.1b2) (Eddy 1998). The candidate MADS-box proteins in this species subsequently verified through annotation in the databases of SMART (Simple Modular Architecture Research Tool), Pfam, and CDD(Conserved Domain Database) using InterProScan and then checked manually to confirm the reliability (Jones *et al.* 2014).

### Phylogenetic construction

The encoded MADS-box sequences from *L. cubeba* and *Arabidopsis* (Huala *et al.* 2001) were aligned using Clustal W. Then, the alignment was refined and removed from the less conserved regions, leaving a conserved MADS domain of 58 characters. NJ (Neighbor-Joining) trees were generated using the MEGA6 program (Tamura *et al.* 2013).

### Motif and gene expression analyses

MEME (Multiple Em for Motif Elicitation) was used to determine the conserved motif of the amino acid sequences in *L. cubeba*, and the parameters of the analysis established as follows: number of motifs at 10, motif width between 6 and 200 (Bailey *et al.* 2009). The identified motifs were annotated using SMART online (http://smart.embl-heidelberg.de/) (Letunic and Bork 2018). The expression of LcMADS-box genes in the flower bud developing stages was generated using Pheatmap in the R software (3.5.1) with data normalized of log2(FPKM+1).

### RNA isolation and cDNA synthesis

The frozen flower organ samples were ground into powder in a mortar with liquid nitrogen, and then, the total RNA was extracted using an RNAprep Pure Plant Kit (Tiangen, China) following the manufacturer’s instructions. The cDNA synthesized from 1 µg of total RNA with a GoldenstarRT6 cDNA Synthesis Kit (Tsingke Biological Technology, China). The final cDNA products were diluted 1:10 with nuclease-free water for reverse-transcription quantitative real-time PCR (RT-qPCR).

### Reverse-transcription quantitative real-time PCR

RT-qPCR amplification was performed in 20 µL mixtures consisting of 10 µL of 2× SYBR Premix Ex Taq II mix (Takara), 0.4 µL each of forward and reverse primers, 0.4 µL of Rox dye, 1 µL of cDNA template (50 ng/µL), and RNase-Free Water. The reaction mixture was incubated at 95° for 30 s; followed by 40 cycles of amplification (95° for 5 s, 60° for 34 s) in the PCR stage; and the melt curve stage was 95° for 15 s, 60° for 1 min, and 95° for 15 s, performed on an ABI QuantStudio 7 Flex Real-Time PCR System. Three technical replicates were run for every sample, and the data were analyzed using the 2^−ΔΔCt^methods (Livak and Schmittgen 2001).

### Transient transformation of LcMADS20 in L. cubeba leaves

To observe the subcellular localization of *LcMADS20*, we cloned the coding sequence of *LcMADS20* and constructed the sequence into the pCambia 1300-GFP/C vector with the cauliflower mosaic virus (CaMV) 35 promoter. The fused plasmid was transformed into *Agrobacterium tumefaciens* strain GV3101 chemically competent cells, which were cultivated in Luria-Bertani medium with 50 mg/L kanamycin and 50 mg/L rifampicin (28°, 200 rpm, 12 h). The strain sediment was collected by centrifugation for 5 min at 5000 rpm at 4° and then suspended in 10 Mm 2-(N-Morpholino) ethanesulfonic acid and 20 µm acetosyringone at OD_600_ = 0.4 and further incubated for approximately 3 h. A needleless syringe was used to infiltrate the leaves of *L. cubeba* without penetration. Finally, the plantlet was maintained in the dark for approximately 2 days and then photographed using a ZEISS LSM 700 laser-scanning microscope at 488 nm to observe the subcellular localization.

### Ectopic expression of LcMADS20 in Arabidopsis

The combined 35S::LcMADS20-GFP vectors and the empty vector (as control) were transformed into *Arabidopsis* via *A. tumefaciens* strain GV3101 using the flower dip method (Clough and Bent 1998).The expression vector contained hygromycin gene, which was useful in verification and screening. The positive transgenic *Arabidopsis*lines were selected on 25 mg/L hygromycin Murashige and Skoog medium and verified using *LcMADS20* specific primers. The seedlings were transplanted into soil cultivated in 7cm *7 cm pot with a 16 h light and 8 h dark photoperiod at 23° until the T3 generation.

### Paraffin section detection of silique thickness

To observe the siliques architecture of the transgenetic *Arabidopsis*, we employed the paraffin section with reference the method of Ye (2012). The silique samples were first fixated in a FAA fixative and then dehydrated on the concentration of 30%, 50%, 70%, 80%, 90% and 100% of ethanol. Subsequently, the samples were embedded into paraffin blocks after treated with xylene, and then cut into thin paraplast sections (6-10μm) using the rotary microtome (ThermoHM325, USA). Additionally, the samples were deparaffinized by xylene and ethanol, and then stained with hematoxylin and eosin solution. Finally, the slices coated with neutral gum were observed using an Olympus BX53 microscope (Olympus, Tokyo, Japan), and the pericarp thickness in siliques were further measured.

### Data availability

The MADS-box genes of *L. cubeba* were analyzed according to data from our previous published paper (He *et al.* 2018). The information of the MADS-box genes of *L. cubeba* was list on the Table S1. The sequences of MADS-box genes of *Arabidopsis* were downloaded from the database of The Arabidopsis Information Resource (https://www.arabidopsis.org/) with the accession number listed on the Table S2. Supplemental material available at figshare: https://doi.org/10.6084/m9.figshare.9978776.

## Results

### Identification and phylogenetic analysis of the MADS-box genes in L. cubeba

**Figure 1:**
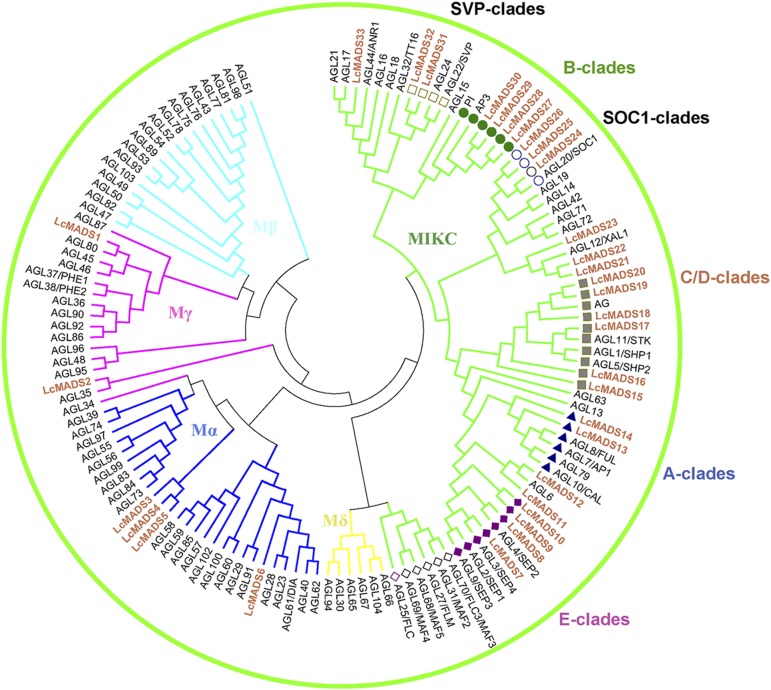
Phylogenetic analysis of MADS-box genes in *L. cubeba* and Arabidopsis. The 33 MADS-box genes in *L. cubeba* (light purple) and the MADS-box genes in Arabidopsis were used to construct the NJ tree based on the MADS conserved domain. The light green branches indicate the MIKC groups, the yellow branches the Mδ, the deep blue branches the Mα, the pink branches the Mγ, and the light blue branches the Mβ. Square indicates the SVP clade genes, circle labeled the SOCI branches, blue triangle labeled the A clades genes, green circle labeled the B clade genes, brown square labeled the C/D clade genes and purple diamond labeled the E clade genes.

A total of 33 LcMADS domain sequences were obtained from RNA-seq data of male and female flower buds in three differentiation stages, respectively (Table S1), and the NJ tree was constructed utilizing the conserved domain with *Arabidopsis* MADS-box proteins (Table S2). Based on the *Arabidopsi*s classified groups (Parenicová *et al.* 2003), 27 members were of MADS-MIKC-type, of which two, four, six, and four members belonged to the A, B, C/D, and E class clades, respectively. Three, two, two, and three members belonged to the *SOC1*, *SVP*, *AGL6*, and *AGL12* clades, respectively (Figure 1).

### Conserved motif analysis of LcMADS-box genes

**Figure 2:**
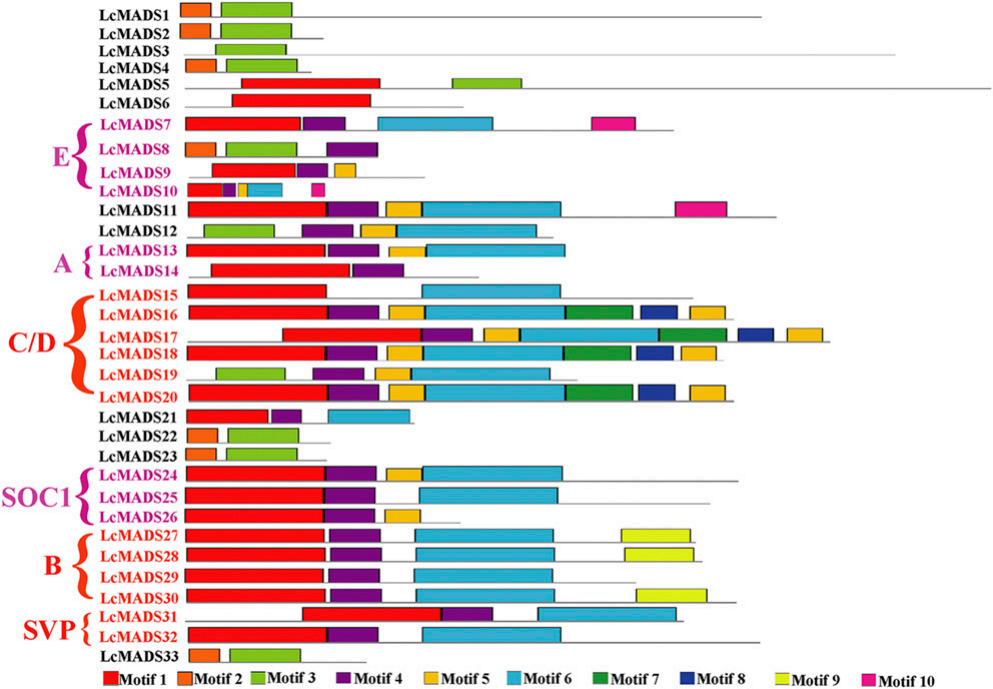
The motif analysis of LcMADS-box genes in *L. cubeba*. Ten motifs were identified, which were filled with different colors. The ABC model genes were labeled. E means the E class genes colored with purple, C/D means the C and D class MADS-box genes colored with red, A means the A class genes colored with purple, B means the B class genes colored with red, SOC1 means the SOC1 clades MADS-box genes colored with purple, SVP means the SVP clades MADS-box genes colored with red.

To identify whether the selected LcMADSs sequences contain the complete functional domains, we conduct the motif searching and clustering analysis for LcMADSs. A total of ten conserved motifs were detected in the 33 LcMADS-box genes (Table S3). Motif 1 represented the typical MADS domains, and motif 6 was the K-box domain that plays a crucial role in protein-protein interaction (Yang and Jack 2004; Kramer *et al.* 1998). The types Mα, Mβ, Mγ, and Mδ all lacked the K-box domain; however, most of the MADS-MIKC type genes had the complete K-box domain (Figure 2).

### Analysis of LcMADS-box genes in flower bud development stages

**Figure 3:**
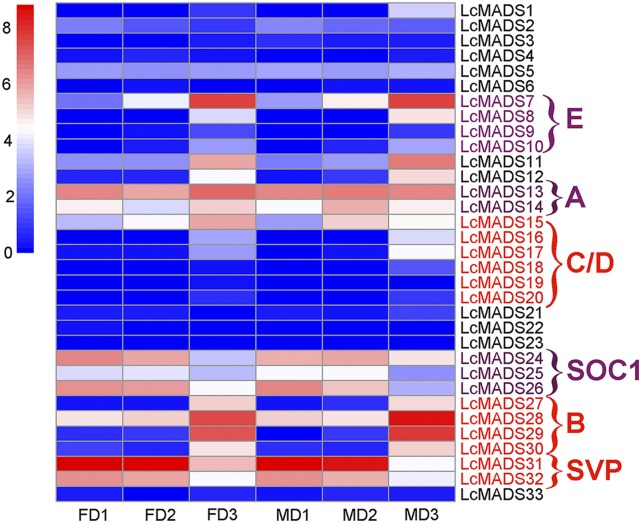
Gene expression of LcMADS-box genes in different stages of flower bud development. The y-axis labels shows the LcMADS-box genes, while the x-axis, including FD1-3 and MD1-3 shows the three stages of female and male flower bud development, respectively. Red means high expression; blue means low expression. The ABC model genes were labeled. E means the E class genes colored with purple, C/D means the C and D class MADS-box genes colored with red, A means the A class genes colored with purple, B means the B class genes colored with red, SOC1 means the SOC1 clade MADS-box genes colored with purple, SVP means the SVP clade MADS-box genes colored with red. The expression data normalized with log_2_(FPKM+1).

Based on the expression patterns in the flower bud developmental stages of *L. cubeba* (Table S4), the A class genes, *SOC1* and *SVP* clade genes all had high expression levels in all three flower bud differentiation stages. However, the expressions of *SOC1* and *SVP* clade genes were relatively highly expressed in the first two stages, indicating that those genes might play a role in the differentiation of inflorescence and flower primordium. Furthermore, the B clade genes, including *LcMADS27*, *LcMADS28*, *LcMADS29* and *LcMADS30*, all of them were highly expressed in the third stages, suggesting that those genes play an important role in flower organ differentiation. The majority of C/D clades genes were constantly lowly expressed in *L. cubeba* flower buds during the different developmental stages. *LcMADS7*, a member of SEP clade genes, had a complete M and K box domains and was expressed consistently with the floral bud differentiation period. *LcMADS8* highly expressed in the third stage, but lower than *LcMADS7* (Figure 3).

### Expression of the C/D class genes in the fruit developmental stages

**Figure 4:**
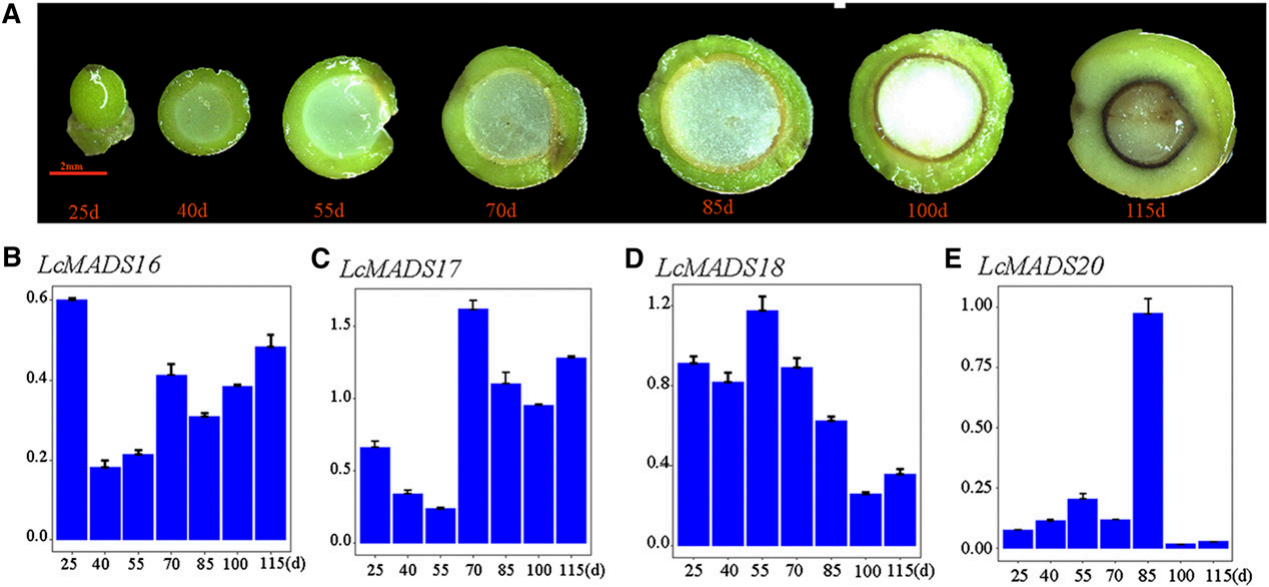
Expression of C/Dclass genes in seven stages of fruit development. (A) The fruit samples used for the RT-qPCR. (B-E) The relative expression of*LcMADS16*, *LcMADS17*, *LcMADS18*, and*LcMADS20*, respectively. The y-axes show the relative gene expression, and the x-axes show the DAF of fruit samples were harvested.

The C class gene AGAMOUS is characterized as a carpel identity gene, and plays a redundant role in controlling ovule identity with other D class MADS-box transcription factors *STK*, *SHP1*, and *SHP2* (Coen and Meyerowitz 1991; Mizukami and Ma 1992; Losa *et al.* 2010). The expression of partial C/D class genes was detected in the developing stages of *L. cubeba* fruits (Figure 4A) using specific primers (Table S5) with the reference gene of ubiquitin-conjugating enzyme (UBC) (Lin *et al.* 2013). The expression of *LcMADS16* was highest in the early fruit development stage (Figure 4B), whereas *LcMADS17* was highly expressed in the middle and later fruit development stages (Figure 4C). The high expression of *LcMADS18* was also likely in the early stages of fruit development (Figure 4D). However, *LcMADS20* was specifically expressed in the middle stage of fruit development, particularly at 85 days after full bloom (Figure 4E) indicating *LcMADS20* play an important role in special fruit development stages. Therefore, we choose *LcMADS20* for further functional investigation.

### Localization of LcMADS20

**Figure 5:**
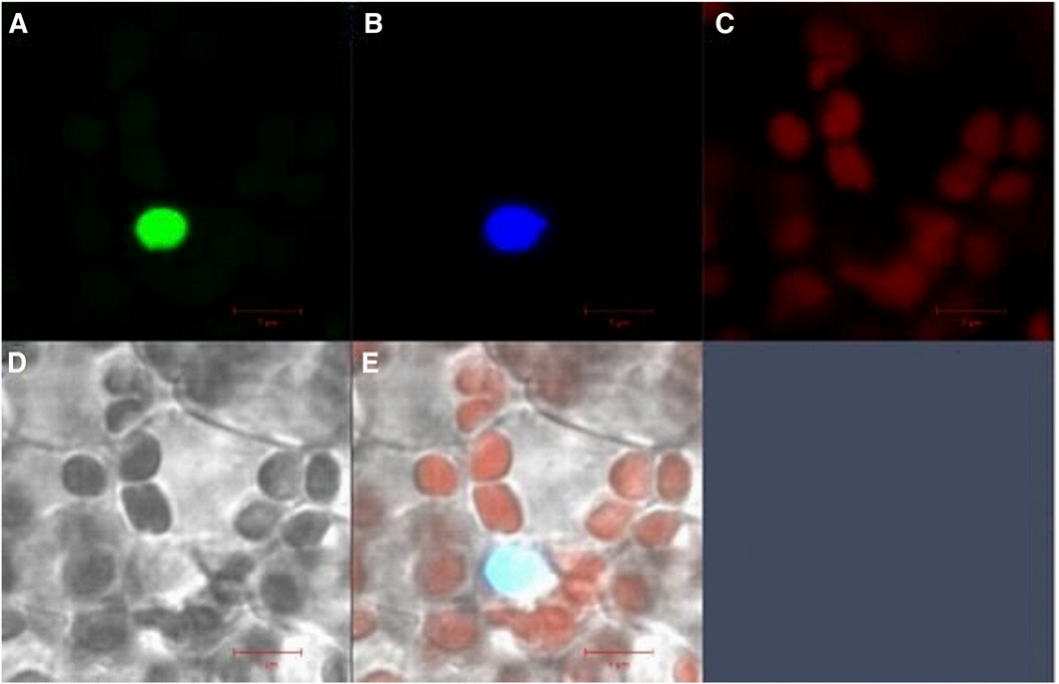
Subcellular localization of 35S::LcMADS20-GFP in *L. cubeba* leaves.(A)The green signal is the GFP fluorescence. (B)The blue fluorescence is the nuclear localization dye of *DAPI*. (C) Red is the chloroplast fluorescence. (D) Bright field. (E) The light blue is the merged fluorescence.

Plant transcription factors consist of a DNA-binding region, a transcription regulation domain and a nuclear localization signal (Liu *et al.* 1999). *LcMADS20*, an AG-like gene, encoded for a 223 amino acid protein with complete M and K domains, and was highly expressed in the fruit developmental stages, especially at 85 days after fertilization. The result suggested that this gene might play an important role in the essential oil production. Therefore, the 35S::LcMADS20-GFP vector was constructed to analyze the potential functions. The experiment of subcellular localization showed that the MADS-box transcription factor, *LcMADS20*, was located in the nucleus (Figure 5).

### Ectopic expression of LcMADS20

**Figure 6:**
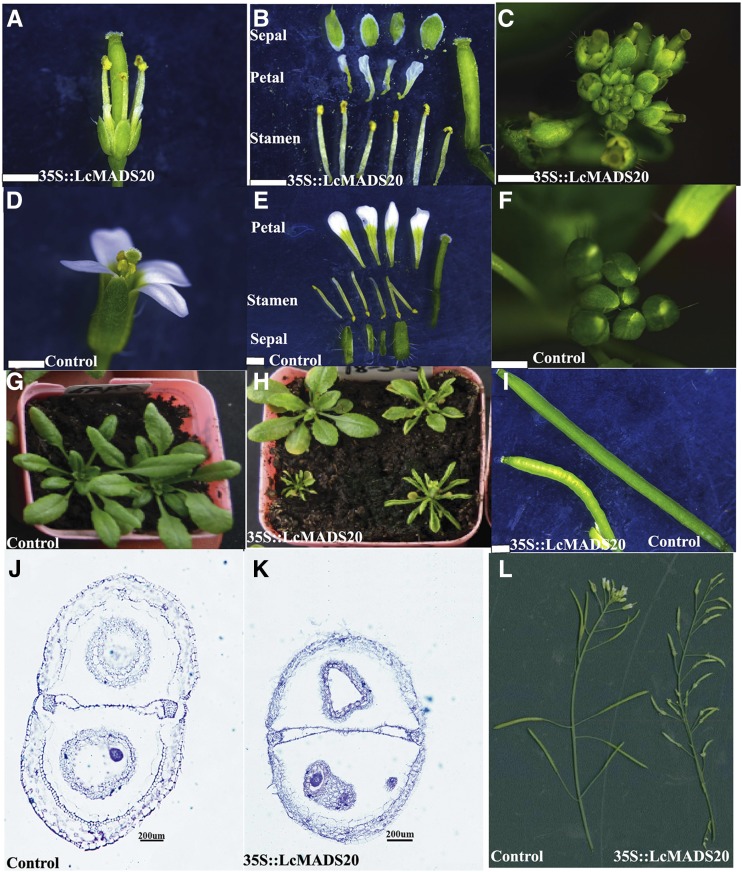
The phenotypes of the 35S::LcMADS20-GFP overexpression *Arabidopsis* and *Arabidopsis* over expressed empty vector. (A) The flower in transgenic *Arabidopsis*. (B) The flower organs in transgenic *Arabidopsis*. (C) Flower inflorescence in transgenic *Arabidopsis*. (D) A single flower in control. (E) Flower organs in control. (F) Normal flower inflorescence in control. (G) Seedlings of control. (H) The seedlings of transgenic Arabidopsis. (I) The twisted silique of transgenic Arabidopsis and normal siliques from control. Bar = 1 mm. The control was *Arabidopsis* overexpressed the empty vector. (J) Paraffin section of siliques in control *Arabidopsis*. (K) Paraffin section of siliques in transgenic *Arabidopsis*. (L) The inflorescence from normal and transgenic *Arabidopsis*.

The combined 35S::LcMADS20-GFP vectors were transformed into *Arabidopsis*, and five positive transgenic *Arabidopsis* lines were acquired (Figure S2). The morphology changes appeared in T_2_ and T_3_ generation compared with the control at the same culture conditions. The severe phenotypes of the over expressed *LcMADS20 Arabidopsis* showed smaller plants, curly leaves and early flowering (Figure 6A-6C, 6H) compared with control (Figure 6D-6G). The curly leaves was similar to the phenotype of both 35S::AP3 and 35S::PI over expressed *Arabidopsis* (Krizek and Meyerowitz 1996). The ectopic expression of *LcMADS20* showed abnormal flowers (Figure 6A) with shorter sepals and narrow petals (Figure 6B), and early opened inflorescence (Figure 6C), which were coincide with the phenotypes of the overexpression of *AG* and *SHP* in *Arabidopsis* (Mizukami and Ma 1992; Riechmann and Meyerowitz 1997; Pinyopich *et al.* 2003). Furthermore, the transgenetic plants showed twisted and shorter siliques compared with control (Figure 6I, 6L) and had similarity with the phenotype of siliques length caused by *ful* and *stk* mutant *Arabidopsis* (Pinyopich *et al.* 2003; Ripoll *et al.* 2015). More of interest, the percentage of pericarp thickness in silique was decreased in *Arabidopsis* overexpressed *LcMADS20*. In detail, the percentage of pericarp thickness in vertical silique was about 9.52% in transgenic *Arabidopsis* comparing with 21.05% in control, and the percentage of pericarp thickness in lateral silique was about 12.50% in transgenic *Arabidopsis* comparing with 37.50% in control (Figure 6J, K).

### The expression of ABC model genes in transgenic Arabidopsis

**Figure 7:**
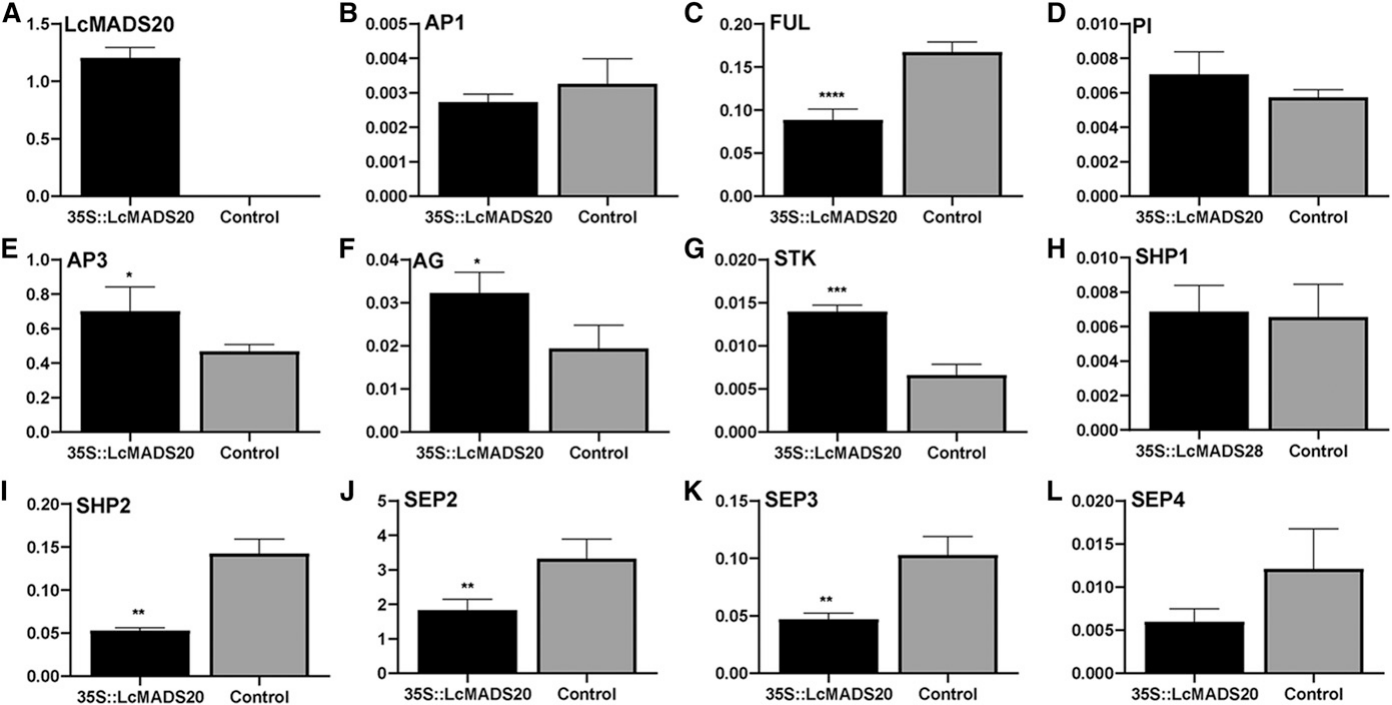
The expression of ABC model genes in siliques tissues in transgenic and control *Arabidopsis*. The y-axes show the gene relative expression level, the x-axes show the siliques materials from *LcMADS20* overexpressed *Arabidopsis* and control *Arabidopsis* (The data statistic was analyzed by one-way analysis of variance, ANOVA).

Overexpression of *LcMADS20* in *Arabidopsis* showed severe phenotypes. However, the plants from the same line showed phenotype changing from slight to severe, even in the T*_3_* generation indicating the expression of flower organ identity genes were affected. In order to verify this assumption, we detected the expression of endogenous ABC model genes using the siliques tissues with the reference gene of actin (Helliwell *et al.* 2006) in *LcMADS20* over expressed *Arabidopsis* and control *Arabidopsis* both from the T_3_ generation (Figure 7A-L), which cultivated at same culture conditions. The results showed the exogenous gene *LcMADS20* was especially highly expressed in the transgenic *Arabidopsis* (Figure 7A). The expression of *FUL*, *SHP2*, *SEP2*, and *SEP3* was significantly decreased in the transgenic *Arabidopsis* (Figure 7C, I-K), while the expression of B class gene *AP3*, and C/D class genes *AG* and *STK*, were significantly increased (Figure 7E-G). The expression of *AP1*, *PI*, *SHP1*, and *SEP4* were shown no obvious change in the *Arabidopsis* overexpressed exogenous *LcMADS20* (Figure 7B, D, H, L). And all the primers used in this paper were shown on the supplementary table (Table S5).

## Discussion

### LcMADS7 was more important in flower bud development stages

The SEP3 clade genes in banana play important roles in peel and pulp development during normal fruit ripening (Elitzur *et al.* 2010). In strawberry, as non-climacteric fruits, the SEP-like gene *FaMADS9* plays an important role in fruit development (Seymour *et al.* 2011). The SEP3-like gene *LMADS3* in lily (*Lilium longiflorum*, a monocot species) ectopically expressed in *Arabidopsis* caused early flowering, reducing plant size, and the loss of floral determinacy, whereas another SEP3-like gene, *LMADS4*, does not cause any change in phenotype (Tzeng *et al.* 2003). In *L. cubeba*, the SEP clades gene *LcMADS7* was highly expressed in the third stages (the flower organ begins to differentiate), suggesting*LcMADS7* may play an important role in flower bud differentiation. Overall, SEP clades may be involved in flower bud differentiation and fruit development.

### LcMADS20 was involved in the flowers and siliques development

Over expression of *LcMADS20* in *Arabidopsis* caused early opened inflorescence, abnormal flowers, short sepals and narrow petals. It has been reported that the overexpression of *AG* and *SHP* can also cause early flowering, curly leaves and prematurely open flower bud (Mizukami and Ma 1992; Pinyopich *et al.* 2003). The results indicated *LcMADS20* might have similar function with the *AG* and *SHP* genes. Correspondingly, the expression of *AG* was significantly increased in the *LcMADS20* transgenic *Arabidopsis*. The phenotype of transgenic *Arabidopsis* showed smaller sepal and petal compare to the control. The sepal and petal identities are generally regulated by MADS-box A class gene (*AP1*) and B class genes (*AP3*, *PI*) in *Arabidopsis* (Bowman *et al.* 1989, Bowman *et al.* 1993; Jack *et al.* 1994; Riechmann and Meyerowitz 1997). However, the expression of the intrinsic *AP1* and *PI* had no obvious change in *Arabidopsis* over expressed *LcMADS20*, only *AP3* was increased compared with control. The phenotype of the short and twisted siliques caused by the overexpression of *LcMADS20* was similar with the silique phenotype of the *ful* mutant (Gu *et al.* 1998; Ferrandiz *et al.* 2000), while *FUL* can activate the miR172 to activate valve growth through the MADS/ARF-miR172-AP2 regulatory module (Ripoll *et al.* 2015). The expression of *FUL* was found decreased in *Arabidopsis* overexpressed *LcMADS20*, indicating overexpression of *LcMADS20* would inhibit the expression of *FUL*.

Overall, the regulation mechanism involved in pleiotropic phenotypes caused by overexpression of *LcMADS20* may be complicated. The floral architecture seems to be not exclusively regulated by single gene or one certain MADS-box class genes. Each class of MADS-box genes is involved in the regulation of flower and fruit development through formation of homo- or hetero-dimer, or other kinds of complexes (Pelaz *et al.* 2000; Smaczniak *et al.* 2012). For example, the AP3/PIheterodimer can interact with *SEP* clades genes to function floral organ identity (Pelaz *et al.* 2000), the MADS-box products can form complexes to control floral architecture (Davies *et al.* 1996; Egea-Cortines *et al.* 1999). Moreover, the function of LcMADS20 should be further investigate in *L. cubeba* in the future. Based on the result, we considered LcMADS20 is a negative regulator involved in the fruits and peel development. The inhibition of LcMADS20 and its regulator will be consider in transgenic *L. cubeba*.

## Conclusion

*L. cubeba* belongs to the Lauraceae family and is a dioecious shrub or small tree. The oil from *L. cubeba* is an aromatic essential oil extracted from the fresh fruits, especially from the peel. It is important to research the MADS-box genes, especially the carpel identity gene, the AGAMOUS-like genes. Thirty-three LcMADS-box genes were acquired in *L. cubeba*, and phylogenetic, motif, and expression pattern analyses were conducted. The A class genes, SVP clade, and SOC1 clade genes were all highly expressed in all the three stages of flower bud differentiation. *LcMADS20* was highly expressed, particularly at 85 days after flowering, which indicated that *LcMADS20* was important in fruit development. At the subcellular level, *LcMADS20* was located in the nucleus. *LcMADS20*, a C class functional gene, can cause curved leaves, early flowering, early opened flower bud, and an abnormal silique morphology in *Arabidopsis*. In *LcMADS20* transgenic *Arabidopsis*, the expression of intrinsic ABC model class genes was influenced, *FUL* was significantly reduced in the transgenic *Arabidopsis*, while *AP3*, *AG*, and *STK* were induced compared with control. The mechanism about how the transcription factor *LcMADS20* and other endogenous MADS-box gene are involved in the regulation of flower and siliques development needs further investigation.
